# Diagnostic accuracy of calretinin and acetylcholinesterase staining of rectal suction biopsies in Hirschsprung disease examined by unexperienced pathologists

**DOI:** 10.1007/s00428-022-03334-3

**Published:** 2022-05-05

**Authors:** L. Beltman, J. D. Windster, J. J. T. H. Roelofs, J. P. van der Voorn, J. P. M. Derikx, R. Bakx

**Affiliations:** 1 Department of Paediatric Surgery, Emma Children’s Hospital, Amsterdam UMC, Meibergdreef 9, 1105 AZ Amsterdam, The Netherlands; 2 Department of Gastroenterology and Hepatology, Amsterdam Gastroenterology and Metabolism, Amsterdam UMC, University of Amsterdam, Amsterdam, Netherlands; 3 Department of Pediatric Surgery and Intensive Care, Erasmus University Medical Center, Sophia Children’s Hospital, Rotterdam, The Netherlands; 4 Department of Pathology, Amsterdam UMC, Amsterdam, The Netherlands

**Keywords:** Hirschsprung disease, Rectal suction biopsy, Calretinin, Acetylcholinesterase, Hematoxylin–eosin, Diagnostic accuracy, Pathologist

## Abstract

Rectal suction biopsy (RSB) is a gold standard for diagnosing Hirschsprung disease (HD). Calretinin staining of RSB is increasingly used by experienced pathologists due to non-complex examination and comparable diagnostic accuracy with acetylcholinesterase (AChE). However, the diagnostic accuracy of calretinin examined by unexperienced pathologists remains to be elucidated. Therefore, we aim to compare diagnostic accuracy of calretinin with AChE on RSB for diagnosing HD when examined by unexperienced pathologists. We prospectively analyzed sections from RSB stained with AChE + HE and calretinin. Blinded examination was done by five unexperienced pathologists (pathology residents) and three experienced pathologists (senior pediatric gastro-enterology pathologists) assessing for the presence of HD. Cases for the study included ones proven to be HD on resection specimens and cases without HD. Diagnostic accuracy was determined calculating area under the curve (AUC), sensitivity, specificity, likelihood ratio, and posttest probability. Fleiss’ kappa analysis was performed to assess interobserver agreement between reviewers. Eleven of 18 included patients (61%) were diagnosed with HD. Comparing the diagnostic accuracy of unexperienced pathologists, calretinin versus AChE + HE showed sensitivity of 80.0% versus 74.5% and specificity of 100% versus 65.4%, AUC of 0.87 (0.78–0.96) versus 0.59 (0.45–0.72). Unexperienced pathologists showed substantial agreement with calretinin (kappa 0.72 [0.61–0.84]) and fair agreement with AChE + HE (kappa 0.34 [0.23–0.44]). We found calretinin having higher diagnostic accuracy in diagnosing HD compared to AChE + HE when examined by unexperienced pathologists. Therefore, we recommend to use calretinin as the standard technique for staining RSB in diagnosing HD.

## Introduction

Hirschsprung disease (HD) is a congenital disorder characterized by abnormal neural innervation of the distal bowel, resulting in obstructive symptoms. The abnormal neural innervation is caused by disturbed migration of neural crest cells during fetal development, resulting in absent or impaired ganglia and hypertrophic nerve fibers [1, 2]. Identification of these characteristics is done using rectal suction biopsy (RSB), the gold standard for diagnosing HD.

The RSB involves suction of rectal tissue above the dentate line. The obtained tissue is processed and stained; whereafter, histopathological examination takes place. The conventional staining method is acetylcholinesterase (AChE) on frozen sections in conjunction with hematoxylin–eosin (HE), which is used in 74% of the biopsies [3, 4]. AChE staining has been shown to be effective at diagnosing HD with a sensitivity and specificity of 97% and 99%, respectively, when examined by experienced pathologists [5]. However, examination of AChE staining is notably challenging for unexperienced pathologists due to the difficulties in distinguishing immature from mature ganglion cells as well as the skill it requires to detect hypertrophic nerve fibers [6]. In the search for an alternative staining method with corresponding effectiveness, calretinin was introduced in diagnosing HD [7, 8]. The use of calretinin shows to be effective when examined by experienced pathologists with a sensitivity and specificity of 96% and 100%, respectively [9]. Under normal conditions, calretinin is expressed by ganglion cells, as well as nerve fibers throughout the submucosa, muscularis mucosae, and mucosa. In the aganglionic bowel, all these structures remain negative for calretinin, resulting in a “black & white” staining pattern which makes the diagnosis of HD less challenging. In addition, calretinin immunostaining is performed on paraffin sections, which have better morphology compared to the frozen sections which have to be used for AChE staining. Therefore, calretinin is expected to be a more suitable staining for RSB when examined by unexperienced pathologists. However, this has not been studied before, concluding that we currently do not know what staining is best to use when an unexperienced pathologist examines the RSB for diagnosing HD.

Therefore, this prospective consecutive case series aimed to find which staining (calretinin or AChE + HE) has the highest diagnostic accuracy, using STARD criteria, when examined by unexperienced pathologists.

## Methods

### Patient population

Twenty-one patients, suspected for HD based on clinical symptoms and radiology examination, were prospectively included from 2012 to 2014. All patients underwent RSB in one of two academic hospitals in Amsterdam (the Academic Medical Center and the VU Medical Center). Exclusion criteria were patients with prior gastro-intestinal anomalies, RSB lacking staining following protocol (AChE, HE, and calretinin), and patients that did not provide informed consent. The gold standard for patients positive for HD was based on histopathological examination of the surgical resection specimen using HE staining examined by senior pathologists with more than 10 years’ experience in pediatric gastro-enterology. The gold standard for patients negative for HD was when patients did not require bowel management treatment during the follow-up in the outpatient clinic.

### Slide preparation

From the included patients, three biopsies were obtained by experienced pediatric surgeons using a rbi2 Suction Rectal Biopsy System at 2 cm ventrally, and 2 and 4 cm dorsally from the anal verge. The obtained RSB were stained differently (AChE, calretinin, and HE) creating panels containing the following three slides: AChE + HE, calretinin and AChE + HE, and calretinin. The RSB were equally divided between unfixed frozen section technique for AChE staining and formalin fixed paraffin embedded sections for calretinin immunostaining. Frozen sections were stained for HE following a standardized method, using a Tissue-Tek Prisma Automated Slide Stainer. AChE staining was performed manually. In short, frozen sections were incubated with acetylthiocholine iodic acid solution (Sigma) at 37 °C for 105 min, followed by incubation with ammonium sulphate (Sigma) for 1 min, after which slides were rinsed and counterstained with hematoxylin (Roche Diagnostics) for 10 s. Slides were mounted with glycerol gelatin.

**Fig. 1:**
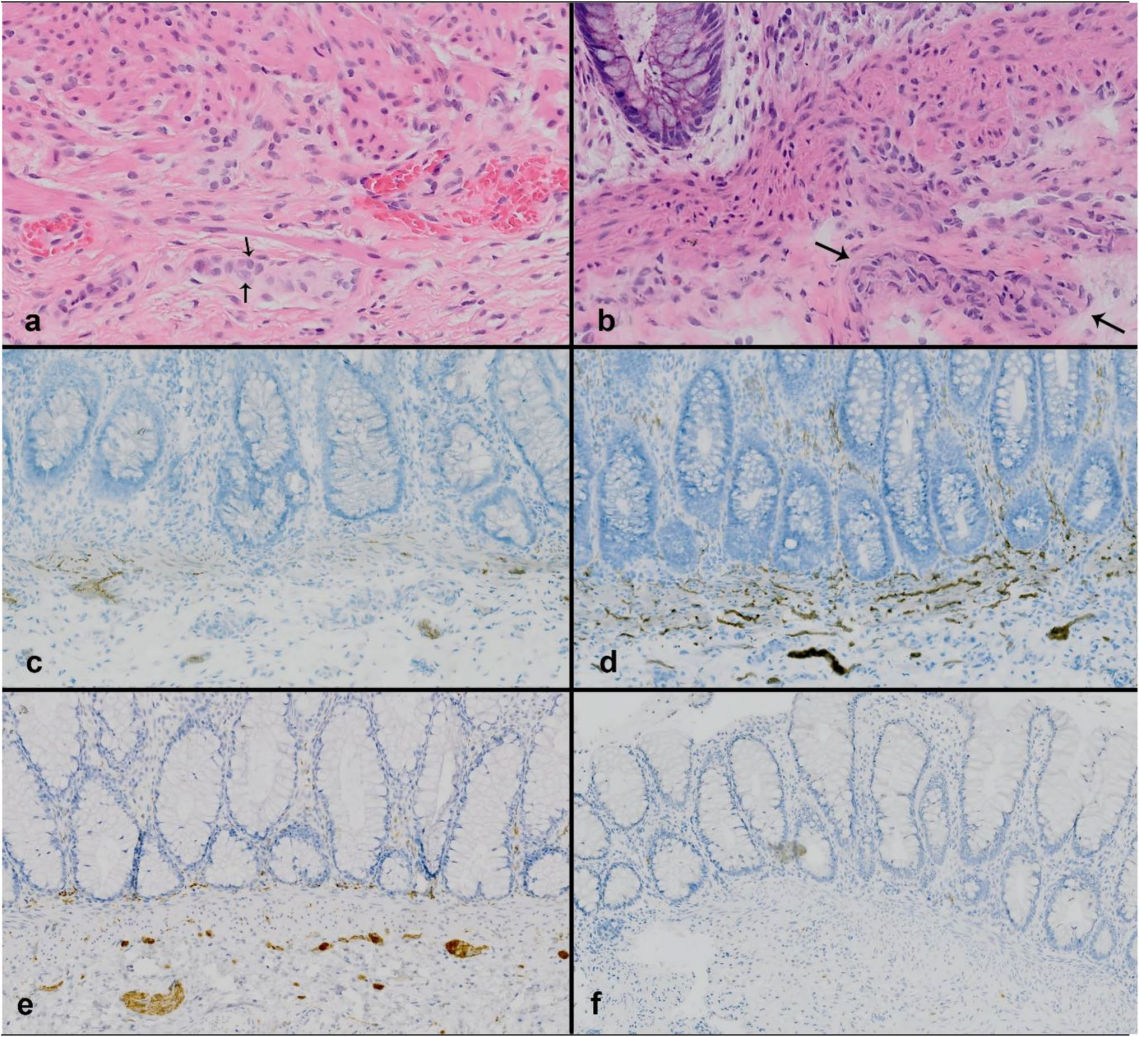
Rectal biopsy sections of confirmed non-HD (**a**, **c**, **e**) and HD (**b**, **d**, **f**) patients. **a** HD negative by HE staining (FFPE section): ganglion cells located in the submucosal region (black arrow), original magnification 20 × ; **b** HD positive by HE staining (FFPE section): in the submucosal region, no ganglion cells are appreciated. The black arrows show hypertrophic nerve fibers, original magnification 10 × ; **c** HD negative by AChE staining (frozen section): some focal positive fibers confined to the submucosa and muscularis mucosa, original magnification 10 × ; **d** HD positive by AChE staining (frozen section): stained fibers are present throughout the breadth of the muscularis mucosa. In addition, positive fibers also in lamina propria, original magnification 10 × ; **e** HD negative by calretinin immunohistochemistry (FFPE section): calretinin positive staining, original magnification 10 × ; **f** HD positive by calretinin immunohistochemistry (FFPE section): calretinin staining demonstrates complete absent immunoreactive fibers, original magnification 10 ×

Paraffin sections were stained for calretinin using a Ventana BenchMark XT autostainer (Ventana Medical Systems, Tucson, Arizona), using a monoclonal rabbit-antihuman calretinin antibody (clone SP13), followed by the appropriate secondary antibody and visualization of positive staining with 3,3′-diaminobenzidine (Roche Diagnostics). Slides were counterstained with hematoxylin (Roche Diagnostics) (Fig. 1).

### Examination of slides

All slides were digitized using an automated slide scanner with 20 × microscope objective (Slide, Olympus, Tokyo, Japan). The digitized slides were anonymized and stored on a secure server. Per patient, one panel was created including the following slides in the configuration (1) AChE + HE, (2) calretinin, or (3) AChE + HE and calretinin. The “Digital Slidebox 4.5” (Slidepath, Leica Microsystems, Dublin, Ireland) virtual slide viewing software was used to examine the digital slides. The examination was done by five unexperienced observers (junior pathology residents) and three experienced pathologists (senior pathologists with ample experience in pediatric gastro-enterology). The training for the unexperienced pathologists for examining the slides was equal for both staining methods and comprised of examination under supervision of an experienced and dedicated pediatric GI pathologist until no interobserver variability occurred. The experienced pathologists received no specific training for the current study, as they had more than 10 years of practice in specialized GI pathology. The unexperienced and experienced pathologists all previously used the digital slidebox platform, and therefore, no additional training for examining digital slides was given. Both unexperienced and experienced pathologists were asked to interpret each randomized panel and indicate if, according to them, it was positive for HD, negative for HD, or not assessable. By doing so, all eight pathologists examined the panels of all included patients, containing three slides per patient, in a blinded fashion. Slides were deemed not assessable to adequately diagnose HD if the quality of the digitized slide was too low for detection of ganglia or thickened nerve trunks, or if the particular slide did not include the muscularis mucosae and submucosa. Slides were deemed positive for HD in case of absent ganglia on H&E staining, combined with increased AChE reactivity of nerve fibers in muscularis mucosae and submucosa, with or without hypertrophic submucosal nerve fibers (diameter exceeding 40 μm). Slides were deemed negative for HD when ganglia were present. With respect to the calretinin staining’s, cases were deemed positive for HD when slides showed calretinin negativity of nerve fibers in mucosa, muscularis mucosae, and submucosa, in addition to absence of ganglia.

### Baseline characteristics

Medical records of all eligible patients were extracted and stored in a Castor database. Validation of the data was done checking 10% of the entered records of each author by another author. In case of an inconsistency, the complete record was checked. The following baseline characteristics were reported: sex (male/female), meconium passage (< 24 h, 24–48 h, or > 48 h), symptoms at presentation (vomiting, distended abdomen, painful abdomen, or other), age at RSB (weeks), length of follow-up (weeks), and length of diseased bowel (short-segment was defined as aganglionosis extending to the rectosigmoid, long-segment as aganglionosis extending to the proximal colon or total colon aganglionosis) [10]. For all the variables, the percentage of missing data was less than 10%. Proportions were reported as percentages. Continuous data was presented either as mean with standard deviation (SD) for normal distributions or as median with range in case of skewed data. Testing the distribution was done using Q-Q plots.

### Statistical analysis

Statistical analysis was conducted using IBM SPSS Statistics for Windows, version 26 (IBM Corp., Armonk, NY, USA). Baseline characteristics were examined using chi-square or Fisher’s exact test for categorical variables and Mann–Whitney *U* test for continuous data. The STARD guidelines were used for determining the diagnostic accuracy of the staining techniques [11]. Therefore, we assessed area under the curve (AUC), sensitivity, specificity, posttest probability, and positive and negative likelihood ratio. Interobserver variability was analyzed using Fleiss’ *k*-statistics for multiple raters with corresponding 95% confidence intervals. Kappa results were interpreted as follows: ≤ 0 as no agreement, 0.01–0.20 as none to slight agreement, 0.21–0.40 as fair agreement, 0.41–0.60 as moderate agreement, 0.61–0.80 as substantial agreement, and 0.81–1.00 as almost perfect agreement [12]. *P*-values of < 0.05 were considered statistically significant.

### Ethics

The Institutional Board of Review approved of this study (W18_160#18.198). All procedures were in accordance with the 1964 Helsinki declaration and its later amendments.

## Results

### Population characteristics

In total, 21 patients were suspected of HD and underwent RSB. Three patients were excluded due to incomplete digital panels (missing AChE slides). No patients were excluded because of not giving informed consent. Consequently, 18 patients were included in our study of whom 14 were male (77.8%) and four were female (22.2%) with a median age at RSB of 5 weeks (range 1–54 weeks) and a median follow-up duration of 235 weeks (range 3–470 weeks). Eleven patients were diagnosed with HD according to the gold standard including eight patients with a short-segment disease (72.7%), one patient with a long-segment disease (9.1%), and two patients with a total colonic aganglionosis (18.2%). From all patients with HD, nine underwent transanal endorectal pull-through procedure and two underwent Duhamel procedure. Patient characteristics including group differences are reported in Table 1, stratified by having HD or not having HD, following the gold standard.

**Table 1:** Patient characteristics (*n* = 18 patients)

	HD (*n* = 11)	Non-HD (*n* = 7)	*P*
Sex, *n* (%) Male Female	10 (91) 1 (9)	4 (57) 3 (43)	.245
Age at RSB in weeks, median (range)	3 (1–26)	11 (5–54)	.050*
Meconium passage, *n* (%) < 24 h 24–48 h > 48 h *Missing*	5 (45) 1 (9) 5 (45) *0 (0)*	4 (37) 0 (0) 1 (14) *2 (29)*	.162
Indication RSB, *n* (%) Vomiting Distended abdomen Painful abdomen Obstruction Other^1^ *Missing*	6 (55) 8 (73) 4 (36) 5 (45) 1 (9) *0 (0)*	2 (29) 1 (14) 2 (1) 2 (1) 0 (0) *0 (0)*	.367 .05* 1.000 .637 1.000
Follow-up period in weeks, median (range)	357 (32–470)	44 (3–246)	.003*

^*^P < 0.05; **P < 0.01

^1^Other indication RSB includes ileus

### Diagnostic value

In total, 144 panels including 432 slides were made wherefrom 88 panels for the HD group including 264 slides and 56 panels for the HD negative group including 168 slides. The unexperienced pathologists examined 90 panels including 270 slides and the experienced pathologists 54 panels including 162 slides. In total, 71 slides were found insufficient (16.4%) wherefrom 35 were examined by unexperienced pathologists (13.0%) and 36 were examined by experienced pathologists (22.2%). The proportion of insufficient slides per staining was: 38 slides for AChE + HE (26.4%), 19 slides for calretinin (13.2%), and 14 slides for AChE + HE and calretinin (9.7%). The results of the diagnostic value of the different staining examined by the total group of pathologists versus the unexperienced pathologists are listed below. AChE + HE showed a sensitivity of 71.1% (total group pathologists) versus 74.5% (unexperienced pathologists) and a specificity of 67.5% (total group pathologists) versus 65.4% (unexperienced pathologists) with an AUC of 0.59 (total group pathologists) versus 0.59 (unexperienced pathologists) and a positive likelihood ratio of 2.2 (total group pathologists) versus 2.2 (unexperienced pathologists). Calretinin showed a sensitivity of 83.3% (total group pathologists) versus 80.0% (unexperienced pathologists), specificity of 98.1% (total group pathologists) versus 100% (unexperienced pathologists), AUC of 0.89 (total group pathologists) versus 0.87 (unexperienced pathologists), and positive likelihood ratio of 45 (total group pathologists) versus not definable (unexperienced pathologists). AChE + HE together with calretinin showed sensitivity of 85.7% (total group pathologists) versus 85.7% (unexperienced pathologists), specificity of 96.2% (total group pathologists) versus 97.0% (unexperienced pathologists), AUC of 0.88 (total group pathologists) versus 0.90 (unexperienced pathologists), and positive likelihood ratio of 22.7 (total group pathologists) versus 28.3 (unexperienced pathologists). Table 2 shows the results of the diagnostic accuracy per staining stratified by the experience of the pathologist.

**Table 2:** Diagnostic accuracy of AChE + HE and calretinin staining examined by unexperienced pathologist (A) versus experienced pathologist (B)

	HD (*n* = 88)		Non-HD (*n* = 56)		Total (*n* = 144)		AUC* [95% CI]		Sensitivity		Specificity		PLR**		NLR***		Posttest probability	
	A	B	A	B	A (*n* = 88)	B (*n* = 56)	A	B	A	B	A	B	A	B	A	B	A	B
AChE + HE							0.59 (0.45–0.72)	0.57 (0.41–0.73)	74.5	60.0	65.38	69.2	2.2	1.95	0.4	0.6		
Positive Not assessable Negative	35	12	9	4	44 (49%)	16 (29%)											79.5%	75.0%
	8	14	8	8	16 (18%)	22 (40%)												
	12	8	17	9	29 (32.6%)	17 (31%)											41.4%	47.1%
Calretinin							0.87 (0.78–0.96)	0.93 (0.87–1.00)	80.0	88.9	100	95.2	-	18.7	0.2	0.1		
Positive Not assessable Negative	36	24	0	1	36 (44%)	25 (45%)											-	96.0%
	10	7	2	0	12 (13%)	7 (13%)												
	9	3	32	20	41 (46%)	23 (42%)											0.22%	13.0%
AChE + HE and calretinin							0.90 (0.82–0.97)	0.83 (0.71–0.97)	85.7	86.2	97.0	94.7	28.3	16.4	0.1	0.1		
Positive Not assessable Negative	42	25	1	1	43 (48%)	26 (47%)											97.7%	96.2%
	6	5	1	2	7 (8%)	7 (13%)												
	4	4	18	18	39 (44%)	22 (40%)											17.9%	18.2%

^*^Area under the ROC curve, **positive likelihood ratio, ***negative likelihood ratio

### Interobserver variability

The total group of pathologists showed a slight agreement on AChE + HE (Fleiss’ kappa: 0.20), a substantial interobserver agreement on calretinin (Fleiss’ kappa: 0.76), and a substantial agreement on AChE + HE and calretinin (Fleiss’ kappa: 0.64). The agreement of Fleiss’ *k* between experienced pathologists and unexperienced pathologists with AChE + HE was slight versus fair (Fleiss kappa: 0.02 versus 0.34), with calretinin almost perfect versus substantial (Fleiss kappa: 0.88 versus 0.72), and with AChE + HE and calretinin both substantial (Fleiss kappa: 0.51 versus 0.68) as shown in Table 2.

## Discussion

The aim of this prospective case series was to find which staining (AChE + HE or calretinin) had the highest diagnostic accuracy when examined by unexperienced observers.

Firstly, we found a high diagnostic accuracy for calretinin staining in diagnosing HD when the slides were examined by unexperienced pathologists. This is in line with the findings of Guinard-Samuel et al. who did not calculate the diagnostic accuracy, but did state that more correct diagnoses were made by unexperienced pathologists when calretinin was used instead of AChE + HE [13]. Presumably, this is mainly caused by the black and white effect of calretinin staining, making objective interpretation easier, and thereby facilitating the challenging diagnosis of HD. In line, we found high interobserver agreement in the group of unexperienced pathologists when calretinin staining was used. This cannot be compared to Guinard-Samuel et al. due to lacking information about the interobserver agreement in their studies.

Secondly, our results show that the sensitivity and specificity of calretinin staining is superior to AChE + HE staining for the total group of pathologists, which is in concordance with the current literature [13, 14]. However, Jeong et al. stated that caution should be exercised when using calretinin for excluding HD because of reporting a lower specificity (85.2% with calretinin versus 100% with AChE) [15]. This is not in line with our results showing a higher specificity with calretinin compared to AChE in the total group of pathologists (98.1% with calretinin versus 96.2% with AChE + HE), experienced pathologists (95.2% with calretinin versus 69.2% with AChE + HE), and unexperienced pathologists (100% with calretinin versus 65.2% with AChE + HE). The higher specificity we found is possibly caused by the small sample size in our study (18 patients and 432 slides) compared to the sample size of Jeong et al. (*n* = 95) [15]. Our sample size may have negatively impacted the number of children with an ultrashort-segment of HD in our study. It has been reported that using calretinin in diagnosing HD in this group of children can lead to false negative results, possibly explaining the higher specificity of calretinin in our study [16]. In our study, we found calretinin leading to false positive results, which was not expected based on previous knowledge. According to Kapur et al., this can be caused by obtaining the RSB not enough proximally for the pectinate line, causing hypoganglionated rectal tissue to be sampled, resulting in false positive results [17].

Thirdly, we compared the outcomes of calretinin only with the combination of calretinin and AChE + HE, finding similar AUC scores in both the total group of pathologists and the unexperienced pathologists. This suggests that there is no added value of combining these staining’s for both groups. Another disadvantage of combining these staining’s is that we found more slides non-assessable when stained with AChE + HE. This can be explained by the staining procedure of AChE, having a more complex staining technique, with broader variations in staining intensities than the black and white calretinin staining’s [15, 18]. Another explanation for this could be the age at RSB; patients who are older at RSB possibly have a thickened mega rectum, resulting in higher failure rates. However, de Arruda Lourenção et al. did not find a significant difference in non-assessable slides between different age groups, defeating this latter theory [19]. The rate of non-assessable slides is of importance because this can lead to a delay that is incurred in making the diagnosis. This can have clinical consequences because we know that a delayed start of adequate treatment can cause severe complications [20, 21]. Therefore, in centers who are unexperienced in diagnosing HD and thereby using AChE, we do not recommend combining calretinin with AChE + HE staining in diagnosing HD, opposed to Jeong et al. [15]. Nevertheless, AChE can be of added value, when prepared and examined by experienced pathologists, since it can provide additional information on hypertrophic nerve fibers when compared to calretinin.

### Limitations

There are some limitations to our study which are mainly related to the limited sample size combined with the fact that a considerable number of slides were deemed not assessable by the pathologists. This might have impacted the validity of our findings despite the balanced distribution of patients with and without HD. We expect the limited sample size to have caused a more heterogeneous group, which has potentially caused discrepancies in our findings. However, all RSB were stained with all three techniques and examined by all observers, therefore expecting the heterogeneous group being of less influence on the outcomes. Next to this, the amount of missing data was limited, only meconium passage having more than 10% missing data. Moreover, we did not collect data on repeat biopsies, whilst this could theoretically influence the diagnostic accuracy of either technique. Another limitation of our study is that the group size of the unexperienced pathologists was not equal to the group size of the experienced pathologists. We deliberately used a bigger group size of unexperienced pathologists in comparison to the experienced pathologists because of the aim of our study. However, the unequal distribution between these groups may have led to a biased result. Furthermore, the gold standard we used for non-HD patients depends on subjective interpretation, potentially leading to false negative diagnosis. However, our long follow-up duration minimized this possible risk.

### Future perspectives

Our findings demonstrate that the diagnostic accuracy of calretinin is superior to AChE + HE staining on RSB when examined by both unexperienced and experienced pathologists in diagnosing HD. Next to this, our data suggest that calretinin staining leads to lower numbers of insufficient biopsies. However, future studies are warranted to investigate if the diagnosis of HD with calretinin staining leads to fewer repeat biopsies and thereby a quicker diagnosis. Therefore, we suggest that future studies should investigate the effectiveness and time efficiency of calretinin staining in the clinical setting, stratified by the examination by unexperienced or experienced pathologists.

## Conclusion

This study describes the diagnostic accuracy using STARD guidelines of both AChE + HE, calretinin, and AChE + HE in conjunction with calretinin, when examined by unexperienced pathologists. We found that calretinin staining of RSB leads to a higher diagnostic accuracy and a higher interobserver agreement compared to AChE + HE when examined by unexperienced pathologists. Furthermore, we found that the use of calretinin leads to a lower number of insufficient biopsies compared to AChE + HE, thereby facilitating faster diagnostic work up. Therefore, we advise the use of that unexperienced pathologist use calretinin as the standard technique for staining and interpreting RSB in the diagnosis of HD.

## Abbreviations

HD: Hirschsprung disease
RSB: Rectal suction biopsy
AChE: Acetylcholinesterase
HE: Hematoxylin-eosin
